# Pesticide retailers’ knowledge and handling practices in selected towns of Tanzania

**DOI:** 10.1186/1476-069X-13-79

**Published:** 2014-10-07

**Authors:** Elikana E Lekei, Aiwerasia V Ngowi, Leslie London

**Affiliations:** Tropical Pesticides Research Institute, PO Box 3024, Arusha, Tanzania; Department of Environmental and Occupational Health, School of Public Health and Social Sciences, Muhimbili University of Health and Allied Sciences (MUHAS), PO Box 65015, Dar es Salaam, Tanzania; School of Public Health & Family Medicine-Faculty of Health Sciences, Anzio Road, Observatory, 7925 South Africa

## Abstract

**Background:**

Approximately 300 pesticide retailers are currently registered in Tanzania. Inadequate knowledge and unsafe handling practices among retailers may contribute to human pesticide exposure and environmental contamination. This study investigated pesticide retailers’ qualifications, work experience, safety practices and the products distributed so as to identify opportunities for preventing Acute Pesticide Poisoning (APP).

**Methodology:**

In 2005, employees of pesticide retail firms in six Tanzanian towns were surveyed using a semi-structured questionnaire and physical inspection of premises. In addition, information on products distributed in 2004 and 2005 was collected from Arusha and Arumeru firms to assess potential risk posed for end-users.

**Results:**

More than half of the participating firms (58.6%) were not registered. Most agents on sale in Arusha and Arumeru were hazardous products including WHO Class I and II products (61.7%) and the mean number of cholinesterase inhibiting agents was 5.8 (range 2–8). Major deficiencies found included semi-trained staff (52%), lack of first-aid kits (38.6%), repacking and decanting of pesticides into smaller unlabelled containers (25.3%), lack of fire-fighting equipment (22.6%) and distribution of unregistered products (9.3%). Compared to unregistered companies, those companies that were registered were more likely to report practicing safe container disposal (40% versus 19%; p = 0.06) and to have an absence of leaking containers (36% versus 15%; p = 0.04).

**Conclusion:**

Pesticide distribution in Tanzania was accompanied by many unsafe practices that may contribute to the burden from APP, not only affecting the distributors but also farmers who buy and use these products. Market pressures appear to be encouraging decanting of pesticides to enable retailers to make profits. Registration of firms appears to be associated with safer practices. Comprehensive interventions to strengthen enforcement mechanisms by increasing the number of pesticide inspectors, ensuring adequate financial support for enforcement activities and providing training opportunities for pesticide retailers and the end users are strongly recommended.

## Introduction

Pesticides retailers in Tanzania are registered under the Plant Protection Act of 1997 [1] and Plant Protection Regulations of 1999 [2]. In 2007, there were approximately 300 pesticide retailers registered in Tanzania to deal with pesticide distribution [3]. The law imposes statutory obligations on registered retailers, including requirements to distribute only authorized products and to maintain safe practices in the handling and distribution of pesticides in order to minimize possible health hazards and environmental pollution.

Retail firms are required to have, at minimum, a technical advisor with competence in the handling of pesticides and knowledge of their health hazards. Such persons are expected to supervise all technical operations on the premises to ensure that pesticides are distributed in a safe manner. In addition, sales personnel are required to have sufficient knowledge about pesticides to enable them to handle pesticides safely and to advise end-users appropriately, which may help to reduce APP incidence and support notification of the agents involved in APP. The influence of pesticide dealers on farmers decisions is well documented worldwide in studies conducted in China [4], South Africa [5], United States [6], Vietnam [7] and Tanzania [8]. Other requirements for registration of a pesticide retailer include the presence of safety equipment, well ventilated premises, fire-fighting equipment, first aid kits and warning signs.

Many countries report common operational problems related to pesticide retail firms. For example, a study conducted in Vietnam reported poor storage, lack of appropriate permits and sale of banned pesticides amongst pesticide retailers [9]. The human and environmental risk from the handing of pesticides at retail outlets is a particular problem in developing countries due to lack of infrastructure [10].

Pesticides may cause serious human health risks not only for users but also for the retailers. A study conducted in the USA between 1998 to 2005 reported that workers employed in two retail industry sectors (farm supply stores and hardware stores) had significantly elevated acute pesticide poisoning incidence rates [11]. Another recent Mexican study reported that pesticide retailers had significantly lower butyryl cholinesterase activity, hemoglobin and hematocrit, elevated platelet count and elevated liver enzyme activity (glutamic-pyruvate transaminase and gamma-glutamyl transpeptidase) and experienced burning sensations in the skin more frequently compared to controls [12]. Similarly, a study in India found the prevalence of gastro-intestinal problems and neurological, ocular, cardiovascular and musculo-skeletal symptoms to be higher among exposed pesticide retailers compared to controls [13].

However, to date, no studies have investigated retailers’ knowledge and practices in countries in Africa. In addition, previous studies conducted amongst the Tanzanian farming community reported that farmers rely on retailers to access pesticides information including use instructions [8] but also suffer high rates of APP [14]. This study therefore investigated pesticide retailers’ qualifications, work experience, safety practices and the products distributed so as to identify prevention opportunities for APP.

## Methodology

**Figure 1:**
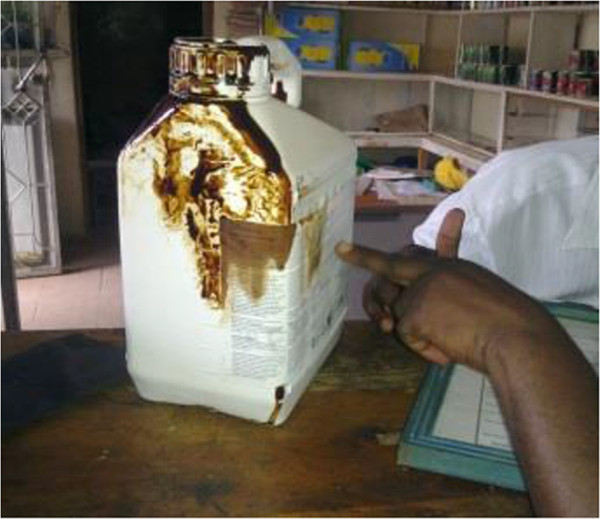
**Inspection and data collection in a pesticides retail firm (repacking of pesticides).**

The population included all pesticide retail firms in the towns of Iringa, Moshi, Kagera, Mwanza, Arusha, Arumeru and Mbeya in Tanzania (n = approximately 200). Assuming a prevalence of appropriate qualifications among the pesticide retailers of 50%, and a precision around the estimate of the mean of 5%, with a total population of 200, a sample size of 132 firms out of 200 firms was chosen. Data from pesticide retailers were collected in a semi-structured questionnaire developed for the study which included predominantly closed-ended and a few open-ended questions that were post-coded. The questionnaire was piloted with a sample of 20 retailers in Arusha town and found to have good face validity and be clear to respondents. Data collected included registration status, staff demographics, storage conditions, the availability of standard safety measures and reported disposal methods. Physical inspection of the premises was conducted to verify the safety features reported and to identify the presence of products that were leaking, repackaged or unlabelled (Figure 1).

In addition, in Arusha and Arumeru information on products distributed by retailers for the period 2004 and 2005 was collected to assess the potential risk facing end-users of these products.

Univariate descriptive statistics were estimated for all variables. For the purpose of bivariate analyses, data were categorized into town location, staff qualification, firm registration status, container disposal practice, container leakage, standard safety requirements, staff work experience as summarized in Table 1. Cross-tabulations using firms as the units were conducted to identify associations with hygiene practices as follows:

**Table 1:** **Data Categorization for Bivariate analysis**

	Variable	Category 1	Category 2
(i)	Town location	**Close**: located close to Tropical Pesticides Research Institute (TPRI)	**other**: located far away from TPRI
(ii)	Staff qualification	**Qualified**: firms with at least one staff member with the TPRI pest management training certificate or a relevant certificate in either livestock, agriculture, health or other relevant science subjects	**Non-qualified**: firms with no qualified staff including staff with form IV certificates, primary school education and qualifications in professions other than science
(iii)	Firm registration status	**Registered:** Firms with an up–to-date permit	**unregistered**: Firms with no permit or out-of-date permits
(iv)	Container disposal practice	**Safe practice**: Burning or bury after washing and puncturing	**unsafe practice**: Dumping in the municipal disposal sites
(v)	Container leakage	**None**: No observed leaking containers	**≥ 1**: At least one leaking container
(vi)	Standard safety requirements	**Available**: Firms with all 6 standard safety requirements available including, Personal Protective Equipment (PPE), first aid kits, fire-fighting equipment, well ventilated premises, display of warning signs and washing facilities.	**Missing**: Firms missing one or more standard safety items
(vii)	Staff cumulative work experience for each firm estimated as total person-years by summing the years staff member worked and then categorizing total person-years for each firm	**Short**: 1–9 years of work experience	**Long**: ≥ 10 years of work experience
(viii)	Presence of unlabelled containers	**None**: No unlabelled container	**≥ 1**: At least one unlabelled container

(i) The variable container disposal practice (safe vs. unsafe) was compared by firm registration status, standard safety requirements, staff qualification and town location.
(ii) The variable container leakage (none vs. ≥ 1) was compared by presence of unlabelled containers, staff qualification, firm registration status, requirements and town location.
(iii) The variable staff qualification (≥1 vs. none) was compared by town location
(iv) The variable presence of unlabelled container (none vs. > 1) was compared by firm registration status.

Statistical testing, using SPSS statistical package version 16 [15] was done by estimating the χ^2^ statistic with statistical significance taken at p < 0.05.

Participants were invited to participate based on full informed consent and assurances of confidentiality. However, participating firms found to be operating contrary to the law were verbally advised accordingly and were provided with official warning letters from the registrar of pesticides after data collection. The warning letters indicated that the registered dealers had to take corrective measures before their annual permit renewal. Firms operating without permits were advised to apply for permits immediately and register their firms.

The study protocol and data collection procedures were reviewed and approved by the Tropical Pesticides Research Institute (TPRI) in Tanzania, the University of Cape Town (UCT) Health Sciences Faculty Research Ethics Committee in South Africa (REF:328/2004) and the Ministry of Health and Social Welfare in Tanzania through the National Institute of Medical Research (REF NIMR/HQ/Vol XI/371).

## Results

The survey involved 75 pesticide retail firms in 6 towns, namely Arusha and Arumeru (n = 15), Mwanza (18), Mbeya (14) Moshi (n = 11), Iringa (Makambako) (n = 10), and Kagera (n = 7). This represents a response rate of 57% of the intended 132 retail firms.

The 75 firms had a total of 175 workers including 40 firm owners; 76% of employees were male (n = 133). The number of workers per retail firm ranged from 1 to 5 and the majority (66.7%) had 1 or 2 workers. At the time of conducting the study, 44% of firms were not registered but reported they were in the process of obtaining registration. Most staff (52%) had either form IV certificates with no additional training (n = 39) or had certificates in general fields without specific pesticide training, such as certificates in agriculture and or livestock handling (n = 38) (Table 2).

**Table 2:** **Academic qualification for the staff working in the visited pesticide retail shops**

No	Qualification	Frequency
1	Degree level (Agriculture, livestock, other)	12
2	Diploma in Livestock, agriculture, other	30
3	Certificates in Livestock, agriculture, other	38
4	Form IV (Ordinary level secondary school certificate) with no additional training	39
5	Standard VII (Primary school certificate) with no additional training	31
6	TPRI Pest Management Certificate	6
7	Diploma or certificate in administration or accounting	18
8	Unreported	1
	**Total**	**175**

Reported work experience varied between 1–5 years (42.6%), 6–10 years (28%), 11–15 years (6.7%), 16–20 years (13.3%) and 20+ years (9.3%).

Pesticides distributed in Arusha and Arumeru by the 15 retail firms surveyed whose records were inspected for details on sales in 2004 and 2005, are listed in Table 3. Inspection found that:

**Table 3:** **Active ingredients distributed by pesticide retailers in Arusha town, 2004–2005**

Pesticide retailer identifier	Total active ingredients	Total WHO I & II	Total OP& CA
A	18	12	8
B	5	4	2
C	17	11	7
D	22	13	7
E	11	7	4
F	24	15	7
G	31	14	8
H	15	11	6
I	22	11	6
J	22	13	7
K	10	7	3
L	8	6	4
M	27	18	8
N	6	4	3
O	26	17	8

(i) The median number of active ingredients per firm was 22 (range 5 to 31).
(ii) The mean number of WHO Class I and II agents on sale was 10.8 (range 4 to 18).
(iii) The percentage of WHO class I and II agents among products on sale in Arusha was 61.7% (Table 3).
(iv) The mean number of cholinesterase inhibitors on sale (OP and Carbamates) was 5.8 (range 2 to 8).

Amongst the products reported as distributed to farmers by the 15 pesticide retailers in Arusha and Arumeru, 47 different discreet active ingredients were identified. Of all products found in the 15 retailers in Arusha and Arumeru (n = 264), active ingredients categorized as OP’s were found most commonly (38%), followed by pyrethroids (n = 35%), Carbamates (32%), Dithiocarbamates (25%), Chloronitriles (10%), inorganics comprising mainly Copper fungicides (16%), Organochlorines (8%), Triazoles (4%), Phenoxy carboxylic acids (3%), Ivamectins (3%) and others (19%). In terms of the WHO Hazard classification system, products distributed were Class I (9.4%), Class II (43.7%), Class III (18.1%) and Class IV or U (28.7%).

Of the 75 firms visited, various deficiencies regarding handling practices were noted. These included employment of one or more untrained sales attendants (i.e. lacking an appropriate qualification) (57.3%), lack of a first aid kit (38.6%), repacking of pesticides (25.3%), lack of fire-fighting equipment (22.6%), unsuitable Personal Protective Equipment (PPE) (14.7%) or no PPE at all (14.6%), pesticide containers with inadequate or absent labelling (14.6%), sale of unregistered pesticides (9.3%), lack of hand-washing facilities (9.3%), sale of expired pesticides (8.0%) and lack of warning signs (6.6%).

Most products with inadequate or absent labelling had been repackaged or decanted and were usually copper-based fungicides (40%) including Copper oxychloride and Cupric hydroxide. However, this group also included a number of OPs such as products containing Pirimiphos-methyl, Profenofos, and Chlorpyrifos, as well as the organochlorine Endosulfan; 40% were WHO Class II pesticides and two cases involved Class I agents.

Only 9.3% of the firms had all 6 standard safety items available (i.e. PPE, first aid kits, fire-fighting equipment, well ventilated premises, display of warning signs and washing facilities). Approximately half of the firms (50.7%) reported availability of 3 or fewer items of PPE. The varieties of PPE reported by 38 retail firms included gloves (n = 35), respirators (n = 29), masks (n = 34), hats (n = 4), long coats (n = 36), overalls (n = 10), gum boots (n = 17) and goggles (n = 6).

Twelve products, which were repackaged or decanted into secondary containers (Table 4), showed signs of spills due to lack of proper seals and damaged containers; these included copper-based fungicides (n = 7), Pirimiphos methyl (n = 1), Chlorpyrifos (n = 2), Endosulfan (n = 1) and Profenofos (n = 1). WHO Class II accounted for 41% of products with spillage.

**Table 4:** **Active ingredients for the products found with substandard labels**

Product active ingredient	Frequency	Repackaged	Chemical group	WHO class
Copper	14	Yes	IN	III
Cynbush	1	No	PY	II
Diazinon	2	No	OP	II
Mancozeb	3	No	OT	IV
Chlorpyrifos	2	Yes	OP	II
Permethrin	1	No	PY	II
Pirimiphos methyl	2	Yes	OP	II
Paraquat	1	No	OT	II
Gammalin(Lindane)	1	No	OT	II
Deltamethrin	1	No	PY	II
Amitraz	1	No	CA	III
Zinc phosphide	1	No	IN	Ib
Lambda Cyhalothrin	1	No	PY	II
Snip (Unknown)	1	No	UN	
Endosulfan	1	Yes	OC	II
Profenofos	1	Yes	OP	II
Carbofuran	1	Yes	CA	Ib
**Total**	**35**			

About half of the retailers reported disposal of waste pesticides in municipal disposal sites (n = 37) and fewer reported disposal of waste pesticides through burning (n = 15) and burying (n = 10). Similar patterns applied to the disposal of empty containers, which the retailers reported disposing of mainly through dumping in the municipal disposal sites (n = 49), burning (n = 13) and burying (n = 4).

Problems appeared to vary by area. For example, in Mwanza and Kagera (n = 25) the most serious problems were the sale of unregistered products (52%), lack of washing facilities (52%) and the presence of semi-trained firm attendants (56%), while in Moshi repacking and decanting of pesticides (36%) was the most frequent concern. The presence of untrained firm attendants was noted in all areas (range 40% to 56%).

### Associations with safe hygiene practices (Table 5)

**Table 5:** **Association with safe hygiene practices**

Practice	Factor associated	p-value ^#^
Safe Container Disposal	Registered firm 40%	0.06
	Unregistered firm: 19%	
	Town location close: 44%	0.05
	Town location other: 23%	
	Standard safety requirements available: 57%	0.04^*^
	Standard safety requirements missing:28%	
	Long cumulative staff experience: 59%	0.03
	Short cumulative staff experience: 33%	
Absence leaking containers:	Registered firm: 36%	0.04
	Unregistered firm: 15%	
	Absence of unlabelled containers: 17%	<0.01
	At least one unlabelled container: 82%	

^#^Unless otherwise indicated, p values based on Chi-squared testing; ^*^Fishers Exact Test.

“Safe container disposal practices was associated with firms being registered versus unregistered (40% vs. 19%, respectively, p = 0.06), firms located close to TPRI versus other (44% vs. 23%; p = 0.05), long versus short cumulative staff experience (59% vs. 33%, respectively; p = 0.03) and standard safety requirements available versus missing (57% versus 28%; Fishers exact test, p = 0.044). There were no associations between container disposal practice and staff qualification. The absence of leaking containers (compared to ≥ 1 leaking containers) was associated with the absence of unlabelled containers vs. at least one un labelled container (≥1) (17% vs. 82%, respectively; p = 0.00) and with firms being registered versus unregistered (36% vs. 15%, respectively; p = 0.04) (Table 5). There were no significant associations between staff qualification and the following variables: compliance with standard safety requirements, presence of unlabeled containers, container leakage and container disposal practices. There were no significant associations between the presence of un labeled containers and the following variables: firm registration status, standard safety requirements, town location and staff qualification (Table 5)”.

## Discussion

In this study, we investigated pesticide retailers’ qualifications, work experience, safety practices and the products distributed. Our findings suggest important opportunities for action to prevent potential adverse health consequences from unsafe handling of pesticides, given that APP has been found to be a significant public health problem in Tanzania [14].

The biggest problems found among retailers appeared to be eminently controllable through the provision of first aid kits, training of sales personnel, provision of PPE and preventing sale of unregistered pesticides and repackaging. Repackaging appears to be associated with considerable spillage of pesticides, was conducted without appropriate PPE or labelling and involved potentially toxic OPs. This practice generates potential for a high risk of exposure for both the sellers and end-users buying the unlabelled products. Repackaging appears to be driven by price and logistics. Expensive products are not affordable to low-income farmers, as a result of which, farmers prefer to buy small quantities often repackaged into drinking water or soft drink bottles. Large pack units are not only unaffordable for small-scale farmers, but also exceed actual need, given the small size of the farms owned by the majority of small-scale farmers.

Repackaging is also driven by opportunities for greater profit-making by retailers. For example, a 50 kg unit of Cobox 50 WP costs around US$50, equivalent to US$1/kg. A 2 kg unit of the same product retails at US$ 5.4 which is equivalent to US$ 2.7/kg, a mark-up of 170%. The high price for small packages is partly attributable to the costs of the containers and the cost of printing labels, but is also an opportunity to profit. This encourages retailers to purchase big volumes for the purposes of repackaging to smaller units in order to extract high profits even though the equivalent small package is available in the market. The presence of repackaged pesticides on farms in this study (25%) was higher than previously found in Tanzania [16] (11%) and, in contrast to the previous study, involved hazardous cholinesterase-inhibiting pesticides. This difference could be attributable to an expansion of private entrepreneurs [17] encouraged by recent market reforms in Tanzania [18] whose safety practices are difficult to control. In a similar study conducted in Burkina Faso, illegal repackaging of pesticides accounted for 9% of the distributors [19], which is far lower than reported in this study. The difference may be potentially attributable to effectiveness of enforcement in Burkina Faso or the smaller size of the country compared to Tanzania. The Burkina Faso study found that only 14% of surveyed premises had a first-aid kit [19] compared to 38.6% found in this study. A similar prevalence of repackaging by retailers in this study was reported in a study conducted in West Indies [20].

The study indicates differences in the degrees of legal compliance regarding pesticides distribution in the North (Moshi) and Lake zone (Mwanza and Kagera). The most serious problems in the Lake Zone were the sale of unregistered products and lack of safety facilities while in Moshi they were repacking and decanting. This may be explained by the fact that Moshi and Arusha are close to the TPRI and frequently visited for enforcement purposes. Retailers may be discouraged from selling unregistered products in areas subject to inspection but may still take risks on decanting, particularly given the absence of legal repackaging plants in Moshi and the resultant temptation to make huge profits. In the Lake Zone, Mwanza and Kagera towns are not regularly visited by inspectors due to their geographical distance and inadequate funds to support travel for inspection. Dealers thus appeared able to make large profits from unregistered products smuggled across borders and sold very cheaply as no import taxes or company registration fees are enforced. Because the products are already cheap generating a wide profit margin for dealers, there may be less pressure to engage in decanting practices in the Lake Zone.

The distribution of these unregistered products is also widespread in this area because the majority of farmers are not able to distinguish registered from unregistered products. Although the list of registered pesticides is published annually and available at TPRI, it is not accessible to the farmers and pesticide dealers. Training, awareness raising and distribution of the list of registered pesticides to the pesticide dealers and farmers are therefore urgently needed.

Similar studies have found regulatory non-compliance, retail firms with poorly trained staff, sale of unregistered and banned pesticides and lack of PPE and first aid kits in Burkinafaso [19] and Vietnam [9] and identified the sale of unregistered products in Kenya [21, 22]. This suggests that the problem is ubiquitous in developing countries.

The study found that a large proportion of products distributed in the study area (35%) were cholinesterase inhibitors and 52% were WHO Hazard Class I and II products. These findings suggest that farmers as end-users are potentially exposed to hazardous and highly hazardous agents. This finding is consistent with studies in Bangladesh, where 66% of the products found in circulation were WHO class I and 11.2% were WHO class II products [23], in the West Indies [20] and in Burkina Faso, where organophosphates and pyrethroids sold by retail firms accounted for about 65% of the active ingredients offered for sale [19]. Ready availability of toxic agents places farmers and rural populations at risk for APP due to the hazardous nature of products stored and used unsafely in these areas [8]. A recent Tanzanian study reported that among the products found to be involved in poisoning, 42.4% were OP’s and 77.6% were WHO Class I and II [8]. These findings have direct implications for APP prevention through interventions with the distributors of these products.

**Figure 2:**
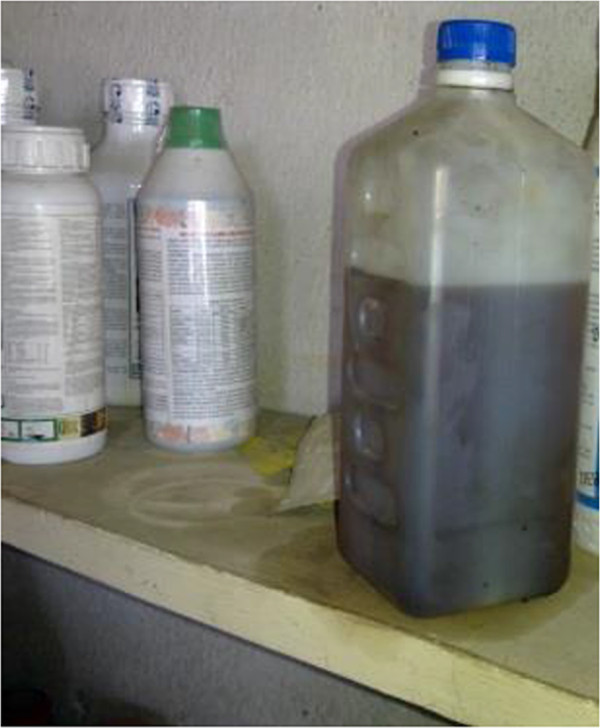
**Liquid products repackaged into a plastic bottle.**

**Figure 3:**
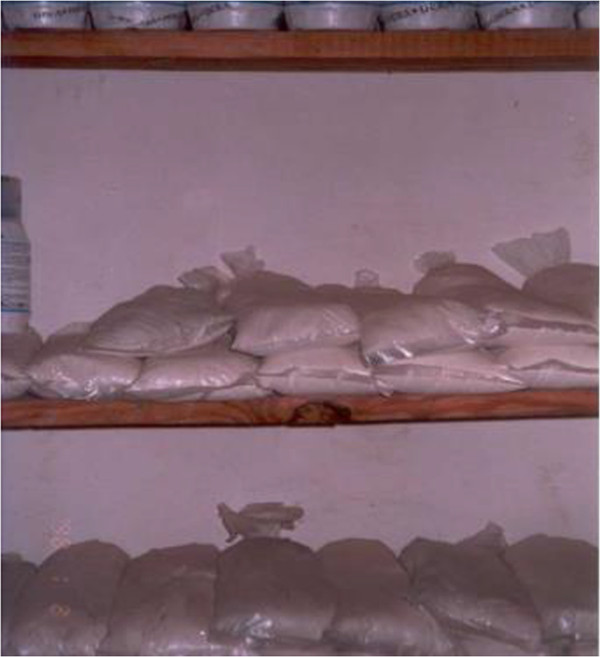
**Powder products repackaged into plastic or paper bags.**

A further concern was the fact that among the products distributed, there were products in unlabelled containers including products repackaged or decanted into containers originally intended for storing drinking water, cooking oil, juices, wine and other liquids (Figure 2). Some dry products were repackaged in plastic or paper bags which resembled bags used for edibles like, sugar or common salt (Figure 3).

Such containers can potentially be mistaken as containing food or beverages and so cause accidental poisonings. They may also be prone to spillage since they can easily break if mishandled during transportation and storage.

The high frequency of unsafe container disposal practice (for example, dumping in the municipal waste stream) among unregistered firms and firms located far away from Arusha could result from inadequate monitoring and absence of guidance on safe disposal methods. Because unregistered firms were not in the TPRI database, they were rarely visited by pesticide inspectors. The association of safe container disposal practice and provision of all standard safety requirements is probably a reflection of a high standard of professionalism in firms seeking to comply with good hygiene and housekeeping practices in terms of the law.

The study indicated that there was no association between having qualified staff and safe practices in pesticide and container disposal. This was an unexpected finding since one would expect trained staff to ensure practice of better hygiene. However, this may result if qualified staff were contracted simply to fulfil registration requirements, after which they were not involved in the actual management of pesticides. Trained staff may well be allocated more demanding and rewarding responsibilities for importation and marketing rather than ensuring safe handling and use, leaving these sensitive assignments to more junior and less qualified staff. These are issues that require active follow-up in inspection and enforcement, as well as improvements in company practice. Lack of training of pesticide retailers and distributors has been reported from a study in Sri Lanka, prompting a recommendation for risk reduction through training retailers in safe handling and storage of pesticides [24].

The study findings suggested a significant association between disposal of pesticides and containers with working experience. The higher proportion of safe disposal practice for pesticides and containers among firms with staff with high cumulative years of working experience could reflect the status of the firms. Larger firms were likely to have more staff, be more likely to be able to comply with regulations and be more professional in their approach than smaller firms. A previous study conducted in Tanzanian [25] reported anecdotal evidence of serious non-compliance amongst pesticide retailers related to selling of unregistered products, repackaging pesticides, selling of expired products, lack of human health safety gear, lack of fire safety gear, untrained firm attendants. However, this study is the first to providing an estimate of non-compliance levels and it is anticipated that these quantitative data will contribute to planning and monitoring appropriate interventions.

Although not the focus of this study, the role of pesticide manufacturers and formulators may be important for safety at the retail level, since they have the capacity, through practicing product stewardship, to promote standard setting for retailers, provide education and information for those supplying their products as well as enable safe storage and return of containers. Responsible stewardship may thus help to improve retailer practices. However, because manufacturers and formulators are located in large urban areas in Tanzania, they were outside the selected study sites and were not included in this study.

### Study limitations

The TPRI is an Institute responsible for enforcement of pesticide legislation, and the researcher’s affiliation to the TPRI as a pesticide inspector may have discouraged participation by unregistered firms and firms with poor safety, as well as deterred respondents from admitting to unsafe practices. To some extent, this potential bias was controlled for by holding a sensitization campaign at the start of the study making participants aware of the study objectives and the research nature of the visit, and assuring confidentiality. For situations where retailers were breaking the law, they were verbally advised to take corrective measures and an official letter was sent to the retailers after data collection to recommend corrective steps. Nonetheless, even if the selection and reporting bias persisted, the effect would have been to result in an overestimate of safety practices.

Further, there might have been some measurement errors unrelated to respondent bias. Companies might have had missing data for unrelated reasons such as poor record-keeping. Further, observation of spilling and unlabelled products could only be done for products on the shelf. Records for poorly labelled products that had already been sold were therefore missed.

However, the likely impact of the different forms of under-reporting would have been to underestimate the extent of poor safety practices in this population so these findings are probably an underestimate of the true scale of the problems existing on the ground. Despite the lack of random sampling, it is likely to be typical of hygiene practices and pesticides distribution in Tanzania.

## Conclusion and recommendations

Pesticide distribution in Tanzania by pesticide retailers is accompanied by many unsafe practices including the sale of products repackaged or decanted in secondary containers, distribution of products with spillage and unsafe disposal of empty pesticide containers. The majority of products distributed by pesticide retailers in Tanzania are highly and moderately hazardous products (WHO Class I and II, respectively). Further, some of the retailers distributed unauthorized products, which had not been tested nor registered in Tanzania. These unsafe practices are likely to contribute to increased risk amongst end-users, affecting not only the distributors but also the farmers who buy and use these products. It also appeared that almost half of the staff working in pesticide retail firms in Tanzania were not properly qualified and hence are unlikely to be able to advise farmers on safe practices. The implication of this unsafe pesticide distribution by retailers, in particular their poor handling practices and distribution of hazardous products, is that the risk of human exposure for farmers buying these products is increased, which may well contribute to APP cases in the community.

Interventions are needed to train pesticide retailers, revise current legislation, strengthen enforcement mechanisms (by increasing the number of well-trained pesticide inspectors and providing adequate financial support for inspection activities) and ensure that appropriately trained technical staff in each retail firm are fully involved in the supervision of pesticides handling, storage and general management in order to reduce human and environmental risks.

There is also an urgent need to provide training on safe handling and use of pesticides to farmers who are recipients of pesticide supplied by the retailers. This is particularly important given that retailers may mislead farmers for personal gain. Training will enable the farmers to be better informed, to monitor retailers’ practice and report non-compliance by the retailers to the authorities.

Regarding repackaging and sale of unregistered products there is a need to emphasize product stewardship such that large companies also take responsibility for non-compliance by small distributors of their products. This form of self-regulation may help to reduce non-compliance among retailers. The knowledge and practices of pesticide formulators and manufacturers would be important for future research so as to plan a comprehensive programme including the upstream factors affecting retailers’ practice. Another potential intervention is the promotion and recognition of retailers’ associations, which can facilitate self-regulation and promote legal compliance for the purpose of reducing unsafe practices. Some retailers’ associations have been reported in Siha district in Kilimanjaro region and the Songea district in Ruvuma region [26] but they need to be formally recognized and supported.

Lastly, it is important that other relevant government officials, such as health officers and municipal law enforcement officials, should be trained to serve as pesticide inspectors. This practice of involving staff from other Government institutions has been reported to work well for medicines and food control under the Tanzania Food and Drugs authority. To ensure that the proposed interventions are effective there is a need for the Government to establish a clear policy across different sectors on health and safety in the distribution handling and use of pesticide in Tanzania.
